# Conceptual Conflicts in Metaphors and Pragmatic Strategies for Their Translation

**DOI:** 10.3389/fpsyg.2021.662276

**Published:** 2021-04-13

**Authors:** Ilaria Rizzato

**Affiliations:** Department of Modern Languages and Cultures, University of Genoa, Genoa, Italy

**Keywords:** conceptual conflict, metaphors, pragmatics, English-to-Italian translation, translation studies

## Abstract

This article seeks to provide a theoretical exploration of Prandi's model of conceptual conflicts in metaphors (2017) and to highlight the advantages such model presents in its applications to translation and the text analysis preceding and preparing translation. Such advantages are mainly identified in the model aptness to meet the pragmatic requirements of translation, seen as a practice-based, goal-oriented and context-driven activity. These advantages also distinctly emerge from a comparison with the main tenets of the cognitive tradition. The theoretical basis for an understanding of conceptual conflict and its applications to translation are illustrated through the analysis of three brief excerpts from literary texts in English and their Italian translation.

## Introduction

Professional translation is a goal-oriented activity based on strong practical objectives and aimed at concrete and effective outputs (Baker, 2011). Since it involves the interaction of two languages, linguistics is an obvious, major contributor to Translation Studies (Baker, 2011) and given the practical nature of translations as concrete products in contingent, specific communicative situations, pragmatics is at the forefront of the linguistic disciplines Translation Studies look to Snell-Hornby (1995, 2006), House (1997), and Baker (2011). Metaphors are traditionally considered a particularly challenging element in translation (Newmark, 1981; Schäffner, 2004). Thus, in the text analysis that precedes and prepares translation, especially in difficult cases, an approach to metaphor analysis which puts an emphasis on pragmatic aspects of metaphors should be adopted and preferred over approaches that do not foreground a pragmatic method.

This article will seek to illustrate how the notion of conceptual conflict in metaphors (Prandi, 2017) may account for pragmatic aspects in metaphors and hence be a reliable model for the metaphor analysis needed in preparation of translation. This theoretical exploration of the conceptual conflict model is conducted by comparing it to the dominant paradigm of metaphor theorization and analysis offered by Conceptual Metaphor Theory (CMT), based on the dual mapping of source and target domains (Lakoff and Johnson, 1980). Its central tenets will be exemplified through literary texts drawing on the author's experience in English to Italian literary translation practice and teaching.

## Conflictual Concepts in Metaphors: Implications for Translation

Although CMT has revealed the pervasiveness of metaphorical concepts in everyday language and thier importance in shaping human thought and communication, its emphasis on generalizable features of metaphor may present detrimental aspects in its application to translation. Emphasis on universal elements in conceptual metaphors and the identification of similar patterns in conceptual metaphors across languages may play down on culture- or language-specific differences that may make a difference between a quality translation and an unacceptable one. The claim of universality for conceptual metaphor, moreover, may have diminished the role of context and situational circumstances in metaphor interpretation, with negative consequences on translation. Snell-Hornby; Snell-Hornby (1995; 2006) critique of universalist theories in favor of differentiation aims to highlight the risk of underestimating the rendering of significant differences in translation.

A consequence of this tendency toward generalization also lies in the idea that there is no difference between conventional and living (that is, unconventional) metaphors since they originate from the same metaphorical concepts (Lakoff and Turner, 1989). This view has significantly turned attention away from living metaphors and reinforced problematic tendencies in translation theory and practice^1^. Identifying conventional meanings across languages is in fact facilitated by dictionaries, linguistic repertories, databases, and similar tools, which rely on normativity and on recurrence in use. Conversely, translating unconventional elements not only requires knowledge of norms, but also sensitivity to deviation from such norms and to possible ways of recreating deviation in the target text with comparable effects. Hence, a model for metaphor identification and analysis encompassing unconventional elements as well as conventional ones may represent a precious tool for translators. This is precisely what Prandi's theory of conceptual conflict does and the reason why this article seeks to explain its main principles and their applicability to translation.

The central notion in Prandi's theoretical framework is that conceptual conflict arises in complex meanings presenting conceptual relations inconsistent with our shared system of conceptual presuppositions or natural ontology (Prandi, 2016: 73–81). A prototypical example of conflict in conceptual relations is Emily Brontë's poetic line “And winter pours its grief in snow” (2017: 23). The metaphor in this excerpt is inconsistent with our shared conceptual structures, since grief cannot be poured, nor can winter feel grief or dispose of it in the form of snow, nor can grief and snow be easily identified as the same thing. Thus, a conflictual meaning arises that cannot rely on shared uses of language to make sense, but that needs an act of interpretation based on co-text, context, and/or the communicative situation at hand. Under a cognitivist perspective, the conceptual metaphors emotions are liquids (within a person) and seasons are persons may be identified as lying at the origin of this example. These two mappings, however, are insufficient to fully unveil the figurative meaning of this line. emotions are liquids (within a person) may explain quite well the import of an utterance such as “being overwhelmed by grief,” through one of the many instantiations of the conceptual metaphor we share in our everyday use of language, representing grief as a mass of liquid submerging a person way beyond his or her capacity. But “pouring one's grief” is not a shared instantiation of the metaphor, since “pour” does not collocate with “grief” in the first place, which makes it conflictual with our shared representation of grief as a mass of liquid within the body or submerging the body. The personification of winter, moreover, may serve to present it as a human being performing actions or feeling emotions, but “pouring grief” is hardly a human action to be performed, nor does the phrasing “its grief” in this context unambiguously express an emotion felt by winter rather than an inner characteristic of this season. Grief is also said to be poured “in snow,” which is not a consistent representation of snow under existing conceptualizations. These elements are inconsistent with our habitual conceptualizations of and presuppositions about them and from this inconsistency conflictual concept arises. Moreover, put together in this context, these elements provide a unique, complex expression that unleashes its figurative and creative potential through an additional interpretative effort on the part of the reader, which is something that with shared conceptualizations does not apply. Being aware of this is a valuable resource for the translator, both for the interpretative act triggered by the metaphor to make sense of it and for the creative act required to translate it in the target language: conflictual elements will have to be identified as such rather than referred back to pre-existing conventional elements, and solutions that render this conflict (rather than relying on conventional conceptualizations) will have to be found.

Conventional metaphors, on the other hand, do not feature conflict since they are consistent conceptual structures belonging to a shared heritage of everyday expressions, emerging from polysemy (Prandi, 2017: 23). An example of this is provided by “wasting time.” The verb “waste” is polysemous and when used with “time” it appears in its metaphorical sense, consistent with the underlying metaphorical concept time is money. The latter concept is part of a shared way of representing time as a valuable resource, already existing in our vocabulary and system of communicative options. Hence, there is no conflict between the idea of “wasting” and the concept of “time.” No particular interpretative effort is required to make sense of the phrase, since its meaning is already conventionalized in our shared linguistic background. In other words, to make sense of such metaphors, one only has to master shared conceptual structures and lexical systems. This has obvious consequences in translation: on the one hand, smaller effort is required to understand what is conventionalized in a shared lexical system, especially in the typical professional translation situation, with a non-native (although usually near-native) speaker of the source language translating into their native language. When concepts and lexis are already shared in a language, they will be immediately accessible to language users or retrievable through dictionaries, corpora, databases, and similar sources. The translator will be aware whether that concept is already available in their native language, and select the conceptual framework that is more apt to translate that metaphor in that context. As emphasized by the cognitivist tradition, conceptual metaphorical frameworks will often go beyond the boundaries of individual languages, making the translator's task theoretically simpler, especially when closely related languages are under analysis. Going back to Prandi's example time is money, it is true that il tempo è denaro provides a nearly identical conceptualization in Italian, so that its projections “wasting time” and “saving time,” for example, will easily be translated, with the necessary adaptations to co-text, through “sprecare tempo” and “risparmiare tempo,” respectively. “Spending time,” on the other hand, is not effectively translated by “spendere tempo,” which may be occasionally found in Italian usage, mainly as a calque from English. The shared Italian equivalent in use will be “passare/trascorrere del tempo,” which does not pertain to the same conceptualization, and using “spendere tempo” in its place will sound marked and unconventional, if not simply awkward. This is a typical case of anisomorphism, which introduces an element of arbitrariness in the shared metaphorical motivation (Prandi, 2017: 186–188). A similar phenomenon warns us that little differences play an important role in a translation effectiveness and that attention to them should not decrease in view of metaphorical pattern similarities between source and target language.

Awareness of anisomorphism also points to the importance of the distinction between non-conflictual/conventional metaphors and conflictual/living metaphors in translation. With conventional metaphors, the metaphor lies in polysemy, that is, in lexical meaning, and the major difficulty lies in translating polysemy itself (as is the case with “spending time”). Conversely, with living metaphors, the metaphor is not translated—what is translated is conflict, and it is up to the reader to interpret it. Text examples in support of this claim are provided in the next section.

Prandi's model also explicitly highlights the importance of the pragmatic level of figurative interpretation in metaphors. In the presence of an extended sense of polysemous word—as in “*wasting”* time —metaphor is logically independent of interpretation, since the metaphorical meaning of “wasting” belongs to the shared lexis of English, and if it occurs in a sentence, it contributes to its complex meaning like any other lexical meaning. In the case of consistent metaphorical expression such as Dante's “In the middle of life's road/I found myself in a dark wood,” the conceptual content of the metaphor is not the outcome of an act of interpretation, but part of our shared conceptualization of life as a journey. Hence, if only conventional metaphors are taken into consideration, there is no interpretative meaning specific to metaphor and its comprehension functions just like any other act of understanding, which is underlined by Sperber and Wilson (2008: 84). In the presence of conceptual conflict, on the other hand, the relationship between meaning, interpretation, figure, and message takes on a specific shape. This is due to two main reasons: firstly, a conflictual meaning lacks conceptual consistency, which imposes an act of figurative interpretation; secondly, with conflictual meanings the process of contingent interpretation not only connects a complex meaning and a message, but also gives shape and content to the figure. A living metaphor is not encapsulated within the meaning of the conflictual expression. A specific level of figurative interpretation becomes necessary, the relevance of which is explicitly excluded by Sperber and Wilson (2008) and (Prandi, 2017: 255–256). Conflictual metaphor may be open to many interpretative paths, not necessarily going in one, identifiable direction. A shared metaphorical concept may also be involved in its structure. The distinctive element, however, is that “the context plays the active role of urging the addressee to infer unexpected projections that go beyond conventional mappings. In such cases, the pressure of conventional coherence and relevance really turns into creative energy” (Prandi, 2017: 257). Thus, figurative interpretation is exclusive of conflictual metaphors and participates in their making, which distinguishes them from conventional metaphors and is extremely relevant to their translation, as the examples in the next section will hopefully clarify. These distinctions are major differences between Prandi's and CMT's views and shift the focus of attention from conventional metaphors to unconventional ones. Given the more complex nature of conflictual concepts as opposed to non-conflictual ones, further structural entities are required to explore conflict that go beyond the dual model of source and target domain as conceived by Lakoff and Johnson (1980). These notions provide an effective description of consistent concepts because they organize whole conceptual areas in conventional metaphors (Prandi, 2017: 29). For example, in the metaphorical concept life is a journey, “life” and “journey” are not confined to individual sentence level, but feature as labels for whole conceptual domains. The theory of conceptual conflict, on the other hand, extends and puts an emphasis on the analysis of living metaphors, that is, on individual complex expressions and complex meanings, which are characterized by further structure and thus require further notions for a thorough description. For these notions, Prandi draws on Black theory 1954—frame, focus, and subsidiary subject—and on Richards (1936)—tenor. *Frame* and *focus* are the immediate constituents of a conflictual complex meaning. The *frame* is characterized by its coherence with the ongoing text or discourse, whereas the *focus* introduces an incoherent and typically inconsistent concept: for example, in “the moon smiles,” “the moon” is consistent with the communicative situation—the nocturnal setting in Blake's poem—whereas *smiles* is the strange, inconsistent element. Frame and focus are overt constituents of a more complex conceptual structure that also includes covert constituents. The latter are represented through the notions of *tenor* (Richards, 1936) and *subsidiary subject* (Black, 1954), which identify the possible covert counterparts of frame and focus, respectively. For example, in “the moon smiles,” the focus “smiles” acts as a subsidiary subject for its covert tenor, say, “glittering,” whereas the covert element emerging from the frame is the human being, which acts as a covert subsidiary subject on the tenor “the moon” in this specific metaphor (Prandi, 2017). Thus, implicit meanings are elicited, which should also be conveyed in translation.

Prandi's introduction of his conflict-based distinction between living and conventional metaphors is only a part of a plural consideration of metaphors which shows more pragmatic applications to translation than CMT. In the cognitive tradition, the singular term “metaphor” usually defines a wide range of figures and linguistic phenomena, including metaphors in the strict sense of the word, obviously independent of their conventional or unconventional nature, as well as simile and even metonymy. This also emphasizes similarities among figures with different purposes, implications, effects, and translation outcomes. Prandi's model, on the other hand, detects differences among figures through an accurate typology (2004; 2010; 2012; 2015; 2017) and his monograph *Conceptual Conflicts in Metaphors and Figurative Language* (2017) specifically examines constitutive aspects of different figures and their different impact on meaning construction. For example, the different implications of the mechanism of analogy displayed by simile, as opposed to the workings of conceptual conflict in metaphor, are analyzed in detail, in open contrast with the cognitivist view of simile and metaphor as substantially the same phenomenon (Prandi, 2017: 166–170). This view is also reflected in the ingrained practice of translating metaphors as similes, which Newmark (1981) poses as one of the third best strategy out of seven to “solve the problem” of metaphor translation. Even if Newmark's decontextualized set of translation strategies may no longer be a strong point of reference in Translation Studies, the idea that simile may be a good substitute—and thus a good translation—for a metaphor is still widespread. A more thorough approach to metaphors and their distinction from simile and other figures makes it possible to investigate their linguistic features and communicative functions in text, with obvious benefits for translation, which is always a creative act of a specific text in a specific context.

A further effort in Prandi's taxonomy of conflictual metaphors is the notion of metaphorical swarm (2012: 157–166, 2017: 140–145). A metaphorical swarm is a network of interconnected metaphoric associations revolving around the same conflictual concept. The core conflictual concept generates a set of related conflictual expressions through the mechanism of projection. As Prandi suggests, projection “does not share the conflictual structure of the complex meaning that triggers it but can be completely accounted for from within the structure of consistent thought” (2017: 151). The main example of metaphorical swarm provided by Prandi is based on the conflictual concept light is a liquid substance in Romantic literature: “if light is a liquid, it can *flow in rivers and streams, form waves, drops and waterfalls, ponds and lakes*, and so on” (2017: 143). This example illustrates how projection may apply to a number of interconnected inconsistent expressions, “each of which frames in words one node of the complex conceptual network projected by the seminal conflictual expression” (2017: 143). *Swarm* seems to be the perfect term to encapsulate such a constellation of expressions, since it suggests exactly the right inferences: “unpredictability of time, location and size; high mobility; and uneven density” (2012: 158, 2017: 144).

## Discussion of Text Examples

In this section, the applicability of the conceptual conflict model to a pragmatic view of text analysis and translation will be explored through literary text excerpts. These notions, however, are not specific to literature, but apply to any text type, as Prandi's applications to the realm of science have demonstrated (Prandi, 2013). The first example is from Sidney's *The Countess of Pembroke's Arcadia*, where the character Dametas is said to be “muttering and **champing** as though his **cud** troubled him” (Sidney, 1973: 44). “Champing” indicates the act of noisy chewing performed by animals, and is the inconsistent element representing our focus. Together with “muttering” it reinforces the idea he is grumbling his complaints in an irate fashion, adding a beastly flavor to it. Such an animal element is confirmed by “cud,” which points to the half-digested food in ruminants, and has no other conventional meaning that could be consistent with the ongoing text. Thus, thanks to co-textual elements, covert elements emerge: behind “champing,” which is both focus and subsidiary subject, is a covert tenor- Dametas's beastly ruminating of thoughts and worries—and behind the tenor Dametas is the subsidiary subject of a ruminant. This interpretation is not only consistent with immediate co-text, but also with the wider context in which Dametas is repeatedly referred to as coarse and uncouth, and such a context reinforces it. It should be noted that an analysis based on source and target domain only would explain this as an instantiation of the thought is food conceptual metaphor, but that would provide no access to the covert meanings responsible for the effects pursued by text. Identifying the covert elements in text is very important to attempt a translation that preserves these complex meaning relations as well as the comic intention at the heart of this passage. A few major problems, however, arise due to anisomorphism in English and Italian: there is no Italian verb for “champing” that is specific to animals, and thus may act as a focus, apart from “ruminare” which has a slightly different meaning, and also the conventional figurative meaning of “ruminating” intended as “thinking”; neither “cud” has any equivalents that are specific to animals; and translating “troubled” requires a more specific solution in Italian. After long research, I would propose this version: “mugugnando e ruminando come se il fieno gli fosse rimasto sullo stomaco,” which may be backtranslated as “muttering and ruminating as if his fodder lay heavy on his stomach,” where “fieno/fodder” makes reference to his animal nature unambiguous, thus selecting the animal—therefore conflictual—meaning of “ruminare.”

The accurate description of conflictual metaphorical structures provided by Prandi through the notions of frame, focus, tenor, and subsidiary subject takes into account aspects of fundamental importance for a pragmatic view of translation, such as implicit meaning, context-relatedness, the salience of world knowledge and inherent complexity. These factors all play a part in making a living metaphor an individual act of linguistic creation of something new, escaping the boundaries of linguistic conventions. In this sense, conceptual conflict is closely related to the mechanisms of conceptual creativity made possible by linguistic expressions, and represents a major resource for expressing innovative contents and projecting new interpretative frameworks onto real-world scenarios.

Innovative concepts, however, may find resistance on their way to translation, and sometimes the problem does not lie in anisomorphism or any other interlinguistic issue, at least not directly. An interesting example concerns the nocturnal setting depicted in William Trevor's short story “Bravado:”

Not many people were about; it was after midnight, almost one o'clock, the widely spaced lampposts casting *pools of misty yellow illumination*. A man walked his dog in Blenning Road in the same blotchy lamplight, the first of autumn's leaves gathering there also. (Trevor, 2004: 73)

“Blotchy” is a strange, inconsistent element as it describes the lamplight as a liquid. The same conflictual concept also features in “pools of misty yellow illumination,” which precedes and prepares “the same blotchy lamplight,” which is a more marked choice than “pools” to project the characteristics of liquids onto light. This marked representation of light is strictly linked to this specific context, as the lamplight appears “blotchy” precisely because of the “widely spaced lampposts” which cast a patchy illumination rather than a uniform one.

Non c'era molta gente in giro; era dopo mezzanotte, quasi l'una, e i radi lampioni proiettavano *pozze di fioca luce gialla*. Un uomo portava a passaggio il cane in Blenning Road sotto la stessa luce pallida, e anche lì si ammucchiavano le prime foglie autunnali. (Trevor, 2009: 64)

The Italian translation maintains the liquid light metaphor in the case of “pools,” “pozze di fioca luce gialla,” where it is interesting to note that misty is rendered as “fioca/dim,” —which is one of the meanings expressed by “misty.” Blotchy, on the other hand, is rendered as “pallida/pale,” which appears to be context-driven as a synonym for “fioca/pale.” This way, however, it does not translate the idea of liquid light forming blotches, which is the most marked—and creative—element in the description of this urban landscape; rather, it erases this outstanding element and replaces it with a substitute simply repeating the meaning expressed by “misty/fioca.” Thus, no conflictual meaning is suggested and consistency is restored at the expense of the peculiar visual input suggested by the metaphorical focus in context. A reason for this choice might lie in the fact that solutions that are as marked in the target text will also stand out and sound “strange,” which may cause a revisor to edit it, or a translator to erase it in the first place to avoid revision. A possible solution features *in quelle stesse chiazze di luce* (which, in back-translation, would read *in the same blotches of light*). This version would foreground the creative image of the “blotches of lamplight,” motivated by the wide spacing of the lampposts mentioned in the previous sentence, producing a patchy light. This interpretation is reinforced by the mention of *leaves gathering there also*, which refers to leaves lying on the pavement within the perimeter of the “blotches” receiving illumination—whereas “luce pallida/pale light” does not seem to draw attention to the pavement, but to the whole space illuminated by the lampposts.

A similar phenomenon characterizes the translation of a passage from the novel *Abela* by a final year student on the MA in Translation and Interpreting at the University of Genoa where I teach:

Suddenly the boys set up an excited shouting, waving their arms and skipping, as *the cloud of noisy red dust that was a bus came bumping* toward them. (Doherty, 2007).

Improvvisamente i ragazzi iniziarono a urlare eccitati, agitando le braccia e saltando, mentre *una vistosa nuvola di polvere rossa, che proveniva da un autobus, si muoveva* verso di loro.

In this translation, “the cloud of noisy red dust that was a bus” is rendered, in back-translation, as “a flashy/huge cloud of red dust, which came from a bus.” This translation offers a simplified interpretation of this figure, deliberately modifying the defining clause after the figure “that was a bus.” Moreover, it turns “noisy” into “vistosa/flashy or huge,” which cancels the synesthetic reference triggered by the sound adjective associated with visual input, by suggesting a consistent association of visual elements only. The conflict implied in the representation of a bus as a bumpy and noisy cloud of dust is leveled down, and this leveling is reinforced by the translation of “came bumping” with “si muoveva/moved.” Again, conflict is underrepresented, although Italian is certainly equipped with the linguistic material to provide a suitable representation of it.

The phenomenon of diminishing marked and innovative elements in translation is well-documented in the literature, and is usually related to a position of inferiority of the translator, to the so-called translator's invisibility, and to publishers' determination to play down on politically conflictual issues (Venuti, 2008). The latter two examples, however, do not seem to justify this explanation, since they do not contain reference to political or sensitive issues. Nor does the linguistic material used present major problems such as wordplay or untranslatable culture-bound elements. The conceptual conflicts in these examples, however, lack conceptual consistency, which is a preliminary condition for a test of coherence and therefore calls for a figurative interpretation (Prandi, 2017: 256). Their translations refuse that lack of consistency and reach coherence by omitting the conflictual elements in favor of a literal, non-figurative solution. This reflects the common habit of considering conflictual concept as a mistake to be corrected rather than an instance of creativity, which has dominated most of twentieth-century—especially Chomsky's—linguistics and is also well-rooted in public opinion. A model of metaphor analysis that takes conflictual concept into account and explains how it works in terms of constructing goal-oriented effects such as Prandi's may well provide a tool to contrast this tendency and a solid basis for the text analysis needed to render conflictual metaphors effectively.

Identifying a metaphorical swarm also provides an asset in the linguistic analysis aimed at translation, since it makes an important textual feature evident, enabling the translator to recognize the network of figurative elements that may be scattered over longer stretches of text, thus paving the way to recreating text objectives and effects in a credible way. In the following dialogue from *The Two Gentlemen of Verona*^2^, for example, a metaphorical swarm emerges in the dialogue between Valentine who, on his departure for Milan, addresses his humorous reproaches to Proteus, and Proteus himself, who refuses to leave Verona to pursue his love for Julia:

PROTEUS […] For I will be thy beadsman,Valentine.

VALENTINE And on a love-book pray for my success?

PROTEUS Upon some book I love I'll pray for thee.

VALENTINE That's on some shallow story of deep love-

How young Leander crossed the Hellespont.

PROTEUS That's a deep story of a deeper love,

For he was more than over-shoes in love.

VALENTINE Tis true,for you are over-boots in love,

And yet you never swam the Hellespont.

PROTEUS Over the boots? “Nay,give me not the boots”.

VALENTINE No, I will not; for it boots thee not.

PROTEUS What?

VALENTINE To be in love […] (Shakespeare, 2005:s3)

In this scene, Valentine introduces the myth of Leander, who would cross the Hellespont every night in order to visit his lover Hero, by comparing his love to Proteus's. This myth offers an opportunity to represent love as a liquid, through the opposition, among others, of “deep” and “shallow,” referring to “love” and “story,” but also evocative of the Hellespont waters, which makes the metaphorical swarm based on the metaphor love is water emerge. The representation of love as a substance is by no means a novelty, like the locative expression “to be in love” presupposes. Here, however, context and reference to myth represent love in a conflictual way, as water, which is unique to this text. Moreover, a network of metaphorical foci consistent with the central metaphorical concept is constructed. An important part of it makes use of the idiomatic expression “over shoes, over boots,” representing a person as immersed with one's feet into some substance (presumably water or mud), metaphorically meaning “expressing reckless continuance in a course already begun” (Anon, 1933: 996). This expression, no longer comprehensible to today's speakers of English, is related to the locative phrase “in love” twice, as it is not used in its canonical form, “over shoes, over boots,” but is split into two, so that “over-shoes in love” describes Leander, whereas “over-boots in love” is associated with Proteus, which seems to condemn Proteus to an even more desperate condition than Leander's. This creative use of the “split idiom” also elicits the exploitation of the domain of footwear to construct projections of the love is water metaphorical swarm providing humorous connection for each character's line in the remaining part of the dialogue. “Boots” is in fact reprised in Proteus' line “Nay, give me not the boots”—an idiom meaning “don't make a laughing stock of me” (Shakespeare, 1969: 4)—and then in Valentine's reply, “it boots thee not,” this time as a verb, an instance of the multiple uses of polysemy characterizing the texture of much of Shakespeare's plays.

Awareness of the swarm of metaphorical expressions and the functions these metaphors have, both in isolation and as an ensamble, may represent a first step toward recreating them in translation. A few difficulties arise in this attempt. First, in Italian “in love” is not translated by an equivalent prepositional phrase, but by the past participle “innamorato,” the locative reference of which is much less transparent. Hence, in order to suggest the idea of love as a substance in which the lover may swim, the translation uses “immerso nell'amore” (immersed in love), which makes the idea of a liquid or a fluid explicit. Second, the idiomatic expression “over-shoes, over-boots” has no immediate equivalent in Italian. No doubt there are translations for the propositional meaning of the expression, but they may not be suitable for constructing a meaningful sentence, nor are they connected with the domain of shoes and boots evoked by the source text and also represented in the following lines. The Italian translation should ideally draw from the same domain and convey “give me not the boots” with an idiomatic expression of the same meaning containing reference to footwear, and “it boots thee not” with a verb semantically related to shoes or boots of the same meaning. Thus, the connection among each character's utterances would be equivalent to that of the source text, and the figurative pattern established in the source text reproduced in the target text. If you consider the single phrases in isolation, solutions may be available presenting near perfect isomorphism with the source text expressions. For example, “calzare” (to fit and, by extension, to be apt) as a translation of the verb “boot” evokes the domain of footwear and means “to suit perfectly,” which could work in this context. Similarly, “esserci dentro con tutte le scarpe” (to be into something over the shoes) is an idiomatic form including reference to shoes (scarpe), which could serve well the purpose of translating “he was over-shoes in love” and, with some adaptations, “you are over-boots in love” and the following “Over the boots?”. And here is where the third problem arises: there seems to be no equivalent for “give me not the boots” in Italian with a similar meaning and a translation of “boots” or some other item of footwear as a focus in the metaphor. Therefore, the penultimate ring in the chain of figures in the same swarm is broken, and the effect of the whole sequence is put at risk. Evidence of this problem emerges from Perosa's translation, where the love is water swarm ends with the translation of “Over the boots?”, and the next two metaphorical foci (“boots” as a noun and then as a verb) are replaced with a pun based on the stem of the verb “dire^3^”, which is unrelated to the preceding exchange, thus diminishing coherence in the dialogue.

Bompiani translation (Shakespeare, 2015) tries to prevent this by identifying an alternative domain to that of footwear that may translate the pair “over-shoes… over-boots” and also cover the utterances “over the boots,” “give me not the boots” and “it boots thee not,” while remaining consistent with the love is water swarm:

PROTEO […] perché io, Valentino, intercederò per te.

VALENTINO Pregando per me su un libro d'amore?

PROTEO Pregando per te su un libro che amo.

VALENTINO Ossia sulla storia superficiale di un amore profondo, come quella del giovane Leandro che attraversò l'Ellesponto.

PROTEO Quella è la storia profonda di un amore ancora più profondo, tant'è che Leandro si immerse nell'amore fino al collo.

VALENTINO Vero, e tu vi sei immerso fino al naso, anche se non ti sei mai bagnato nell'Ellesponto.

PROTEO Il naso? Sei tu a non dovermi prendere per il naso!

VALENTINO No, no. A fiuto, direi che non fa per te.

PROTEO Che cosa?

VALENTINO L'amore […] (Shakespeare, 2015: 25–27)

A source very productive domain in terms of idioms and polysemy was required in Italian that could cover the five footwear-related items in the most similar way possible—translating, for example, an idiom for an idiom, a polysemous lexeme with an equally polysemous one, and so on. In this context, existing metaphor research was taken into consideration and applied to the search for appropriate solutions, so that the domain of the human body, considered the major source domain for conceptual metaphors (Kövecses, 2010: 18), was identified and actually used in the passage to construct a very similar figurative pattern in the target language^4^.

Thus, “fino al collo” (up to one's neck) was selected for “over-shoes,” “fino al naso” (up to one's nose) for “over-boots,” and “prendere per il naso” (pull somebody by one's nose, metaphorically meaning “making a laughing stock fun of somebody”) for “giving somebody the boots.” The element of the nose was then reprised in the next utterance by “a fiuto” (according to one's sense of smell), which is also idiomatic. Here, it modalises the target text equivalent of “it boots thee not,” “non fa per te,” which translates its propositional meaning without including any metaphors of the body. Thus, a number of adjustments were made necessary by the unavailability of Italian immediate equivalents, but the figurative pattern suggested by the metaphorical swarm in the source text was preserved, creating a similar pace in the translation, and making sense of each line in this further sparring match in a lively manner.

This version does not solve all problems. For example, “fino al naso” is neither part of a lexicalized expression together with “fino al collo,” nor is it as idiomatic, but it serves the purpose of constructing a comprehensible crescendo structure relying on the same domain. In addition, in the final part of the passage, a modaliser (“a fiuto”) is added in the target text to justify the presence of a meaningful metaphorical element from the desired source domain. Moreover, the ridiculous, low-brow connotations of shoes and boots, especially when referred to the noble feeling of love, are not completely translated by the notions of nose and neck. The main objectives pursued by the metaphorical swarm in the source text, however, are at least partially achieved in the target dialogue, where making the figurative texture evident conveys extra meaning, sustains dramatic progression and strongly contributes to comic effects.

## Conclusion

Translation is an inherently pragmatic activity, since it needs to tackle context- and situation-related issues and pay attention to minimal cross-linguistic differences which are unique and specific to the text under analysis. In metaphor translation, the model for metaphor analysis provided by CMT does not seem to offer the same range of tools for translation as Prandi's model, since it emphasizes universal and conventional aspects of metaphors, while translation difficulties are usually due to linguistic difference, anisomorphism and unconventional meanings, as the passages analyzed in this article were meant to demonstrate. Prandi's theory of conceptual conflict seems to provide a better analytic model, since it identifies significant distinctions to be realized in translation, and puts an emphasis on living metaphors, revolving around conceptual conflict and unconventional elements. The notion and structure of conflictual concepts offers more specific resources for text analysis and translation than the mapping of source and target domains. In addition, pragmatic aspects specific to figurative interpretation are illustrated that distinguish conflictual metaphors from conventional ones and make readers, analysists and translators aware of the peculiar nature of figurative meaning. Prandi's model also challenges the view of “metaphor” as one phenomenon to encompass other figures, their differentiated objectives and effects. Within this plural consideration of metaphors, the metaphorical swarm stands out as a useful tool to break down complex metaphorical networks and explain their functions, as illustrated by the excerpt from *The Two Gentlemen of Verona*. In conclusion, the adoption of the conflictual concept model for the text analysis preparing translation allows for a more detailed, text- and context-specific notion of metaphors, which offers very useful insights for choosing the translation strategy to be applied and paves the way for an effective rendering of the source text from a pragmatic point of view.

## Author Contributions

The author confirms being the sole contributor of this work and has approved it for publication.

## Conflict of Interest

The author declares that the research was conducted in the absence of any commercial or financial relationships that could be construed as a potential conflict of interest.

## Footnotes

^1^Although many metaphor scholars, including, to mention but a few, Kovecses, Steen, Semino, and Gibbs have looked at living metaphors from a cognitive perspective, they are not analyzed here because the focus of this article is on the model provided by Lakoff and Johnson and on its unparalleled influence and persistence in approaches to analysis and translation.

^2^The Italian translation provided here is the author's work (Shakespeare, 2015). This passage has already been analyzed in two previous writings by the author, dealing with the translation of the play (Rizzato, 2019) and with the specific functions associated with metaphorical swarms in the source text and in translation (Rizzato, 2021).

^3^PROTEO: No, non mi *dire*./VALENTINO E io non lo *dirò*: non ti si *addice*. (Shakespeare, 1990, p. 419) PROTEUS: Don't even tell me that!/VALENTINE: I won't tell: it doesn't suit you.

^4^Perosa had already used the source domain of the human body to translate the first three footwear-related items, but not the remaining ones, thus altering the structure of the swarm completely, whereas Bompiani translation attempts to preserve such structure in the target text as a fundamental item in meaning-making.
